# Severe processing capacity limits for sub-lexical features of letter strings

**DOI:** 10.3758/s13414-023-02830-1

**Published:** 2024-01-03

**Authors:** Maya Campbell, Nicole Oppenheimer, Alex L. White

**Affiliations:** Department of Neuroscience & Behavior, Barnard College, Columbia University, 76 Claremont Ave, New York, NY 10027 USA

**Keywords:** Visual word recognition, Reading, Attention: divided attention and inattention, Attention: theoretical and computational models

## Abstract

When reading, the visual system is confronted with many words simultaneously. How much of that information can a reader process at once? Previous studies demonstrated that low-level visual features of multiple words are processed in parallel, but lexical attributes are processed serially, for one word at a time. This implies that an internal bottleneck lies somewhere between early visual and lexical analysis. We used a dual-task behavioral paradigm to investigate whether this bottleneck lies at the stage of letter recognition or phonological decoding. On each trial, two letter strings were flashed briefly, one above and one below fixation, and then masked. In the letter identification experiment, participants indicated whether a vowel was present in a particular letter string. In the phonological decoding experiment, participants indicated whether the letter string was pronounceable. We compared accuracy in a focused attention condition, in which participants judged only one of the two strings, with accuracy in a divided attention condition, in which participants judged both strings independently. In both experiments, the cost of dividing attention was so large that it supported a serial model: participants were able to process only one letter string per trial. Furthermore, we found a stimulus processing trade-off that is characteristic of serial processing: When participants judged one string correctly, they were less likely to judge the other string correctly. Therefore, the bottleneck that constrains word recognition under these conditions arises at a sub-lexical level, perhaps due to a limit on the efficiency of letter recognition.

**Supplementary Information:**

The online version contains supplementary material available at 10.3758/s13414-023-02830-1.

## Introduction

Reading is integral to our daily lives and is one of the most challenging tasks faced by the visual system. While a full passage of text may be presented to a reader all at once, processing capacity limits on word recognition slow the comprehension of all that information (Reichle et al., 2009; White et al., 2020; White et al., 2019a; Yeatman & White, 2021). A major limiting factor is the loss of high-resolution information as eccentricity (distance from the fovea) increases (e.g., Legge et al., 1997; Pelli et al., 2007; Veldre et al., 2023). As a result, readers must make many short saccades to fixate on words in a sequence (Rayner et al., 2016). But even within one gaze fixation, multiple words can often be perceived, and there has been much debate as to how those words are processed over time. Models of reading either assume parallel processing, in which attention is divided over several words that can be recognized at once (Engbert et al., 2005; Snell et al., 2018), or serial processing, in which attention focuses sequentially on each word (Reichle et al., 2006).

Another line of work has investigated whether people can recognize just two isolated words at exactly the same time (White et al., 2018, 2020; White, Palmer, et al., 2019b). These studies used a “dual-task” paradigm, in which participants view pairs of words that are flashed briefly and then replaced with postmasks that erase the words from iconic memory. In the “single-task” condition, participants are cued in advance to focus attention on just one word that they must categorize at the end of the trial. In the “dual-task” paradigm, the participant is precued to attend to both words, because at the end of the trial they must categorize both independently. (In this context, “dual-task” does not mean that the participant does two totally different tasks; rather, they must make two of the same type of judgment about two stimuli, independently.)

The advantage of this approach is that we can directly compare task performance against the quantitative predictions of three models of processing capacity limits that assume either parallel or serial processing of two stimuli. These models predict how accuracy is affected by attempting to divide attention compared with focusing on just one stimulus (Scharff et al., 2011; Shaw, 1980; Sperling & Melchner, 1978). The serial model assumes that the participant can only recognize one stimulus per trial because they do not have time to start processing the other before the postmasks have replaced it. When the participant is asked to judge the stimulus that they did not process, they can do no better than guess. That causes a severe drop in dual-task accuracy compared with single-task accuracy, of a particular magnitude that the model predicts for each participant. In contrast, two standard parallel models propose that attention can be divided among multiple stimuli with either no cost to accuracy (independent parallel, unlimited-capacity model) or a modest cost (fixed-capacity parallel model). By calculating the difference between the model predictions and each individual’s task performance, we can reject some models in favor of another.

In lexical decision and semantic categorization tasks, accuracy was so much worse in the dual-task condition that it supported the serial model (White et al., 2018, 2020; White, Palmer, et al., 2019b). Moreover, there was a *stimulus processing trade-off* in the dual-task condition: accuracy was worse for one side when the other side was judged *correctly* rather than incorrectly. That is also consistent with the serial model. However, when participants viewed the same sequences of stimuli but judged the color of the letters rather than the meaning of the words, the dual-task deficit was much smaller, consistent with parallel processing of the surface features of the two words (White et al., 2018, 2020). These data imply that an internal “bottleneck” lies between early visual and lexical analysis.

This implication is supported by neuroimaging data that show early stages of visual processing are spatially parallel, with neurons in different parts of retinotopic cortex processing information from different visual field locations simultaneously (e.g., White et al., 2017). Those parallel visual channels appear to converge in the anterior part of the visual word form area, a text-selective region of ventral temporal cortex (White, Palmer, et al., 2019b). The authors concluded that the visual system could process two words in parallel until a late stage in the ventral stream.

One key question is: What types of information about written words *can* be processed in parallel? Here, we used the dual-task paradigm to investigate the capacity limits that constrain two sub-lexical stages of processing letter strings. The first experiment targeted letter identification, a basic building block of word recognition. The participant’s task was to report the presence or absence of a vowel in a string of consonants. The second experiment targeted a higher level of processing: phonological decoding. The task was to report whether the letter string was pronounceable or not. This task could also be described as judging the *orthographic regularity* of letter strings—whether the combination of letters is like those frequently encountered in real words and conforms to the rules of English pronunciation. In both experiments, the stimuli were strings of letters that did not form real words.

## Method

### Experiment 1

#### Participants

Eleven volunteers (seven female, two male, two nonbinary; nine right-handed; age 22.2 ± 4.7 years) with normal or corrected-to-normal visual acuity participated. Participants gave informed consent in accordance with the Declaration of Helsinki and Barnard College’s Institutional Review Board. All were native English speakers and had not been diagnosed with dyslexia or any other perceptual or cognitive disorder.

#### Equipment and stimuli

We used custom MATLAB software (The MathWorks) and the Psychophysics Toolbox (Brainard, 1997; Pelli, 1997) to present stimuli on a ViewPixx 3D screen (VPixx Technologies). Throughout the presentation of stimuli, we recorded the right eye’s gaze position at 500 Hz with an EyeLink 1000+ video-based eye tracker.

The stimuli were presented on a white background (100 cd/m^2^). The fixation mark, present throughout each trial, was a small black fixation cross enclosed in a black circle of 0.38 degrees of visual angle (°) in diameter, with a white dot at its center. The target stimuli were five-character black letter strings in Courier New font, scaled such that the “x” was 0.6° in height. The distance between the centers of neighboring letters was 0.79°, standard for that font at that size. There were two categories of stimuli: *Vowel-absent* letter strings contained all consonants. *Vowel-present* letter strings contained four consonants and one vowel (*a*, *e*, *i*, *o*, or *u*) in any position. All the *vowel-absent* letter strings contained one of the following consonants that previous research has indicated are visually similar to a vowel: *q*, *j*, *c*, *n*, or *s* (Janini et al., 2021). No letter strings were pronounceable, and none contained the letter *y*. Each string contained at least four unique letters. There were 1,651 unique letter strings of each category. Postmasks were strings of five black characters in the BACS-2 serif pseudofont created to visually match Courier New (Vidal et al., 2017).

#### Trial sequence

**Fig. 1:**
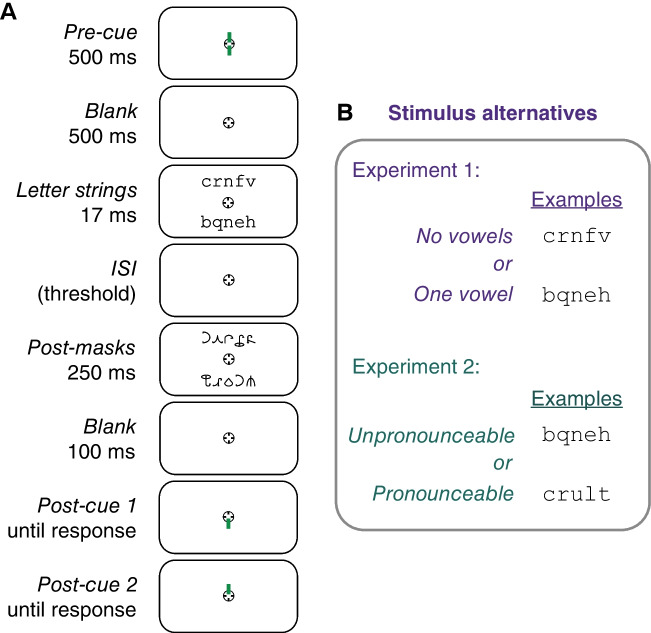
Stimuli and trial sequence. **A** Example dual-task trial sequence. ISI = interstimulus interval. Not shown is the 1,000-ms intertrial interval after the last response on each trial. Single-task trials were identical, except the precue (green line) pointed to just one side (top or bottom), and there was only one postcue at the end of the trial that prompted the participant to judge just the precued side. In Experiment 1, the task was to report whether the letter string on the postcued side contained a vowel or not. In Experiment 2, the task was to report whether it was pronounceable or not (and all letter strings contained one vowel). **B** Stimulus alternatives that the participant had to distinguish between, with examples for the two tasks. (Color figure online)

As shown in Fig. 1A, each trial began with a 500-ms precue. In dual-task trials, the precue was composed of two green vertical lines, each 0.16° long, one above and one below the screen center (inner endpoints 0.05° from the screen center). In single-task trials, only one of those two lines appeared, indicating the side (top or bottom) that would be postcued. After a 500-ms blank interval containing only the fixation mark, two letter strings were flashed for 17 ms. The strings were centered 1.5° directly above and below the fixation mark. Each string was equally likely to be drawn from either of the two categories (vowel present and vowel not present), independently of each other. Thus, the correct response for one stimulus did not predict the correct response for the other stimulus.

The stimuli were followed by an interstimulus interval (ISI) containing only the fixation mark with a duration set to each participant’s threshold for 80% correct with focused attention (for details, see Procedure). The ISI was the same duration in single-task and dual-task trials. Following the ISI, two postmasks were shown for 250 ms, centered at the same locations as the letter strings.

After an interval of 100 ms containing only the fixation mark, the postcue appeared. In single-task trials, the postcue was the same as the precue, indicating which stimulus the participant should judge. At 500 ms after the postcue, a beep prompted participants to respond. As soon as the subject pressed a response key, a 100-ms feedback tone was played: low pitch (180 Hz) for an incorrect response, or high pitch (600 Hz) for a correct response.

Dual-task trials were identical to single-task trials, except for the precue (which pointed to both sides) and the postcues, which prompted the participant to judge both letter strings in random order. After the postmask, the postcue pointed to one side (randomly selected), and the participant responded about the stimulus on that side. After 300 ms, the postcue reversed and pointed to the opposite side, and another beep prompted the participant to give their second response. Two feedback beeps were then played: one for the first response and one for the second response. The next trial began after a 1,000 ms intertrial interval (ITI).

The task was vowel detection: to report whether the postcued letter string contained a vowel or not, along with a report of confidence. The subject pressed one of four keys (m, <, >, ?) with their right hand when the postcue pointed to the bottom side, and pressed one of four keys (*a*, *s*, *d*, *f*) with their left hand when postcued to the top side. For each hand, the left-most key corresponded to “sure vowel absent,” and the right-most key to “sure vowel present.” The middle two keys corresponded to “guess vowel absent” and “guess vowel present,” respectively.

#### Procedure

Participants first received instructions, practiced the task with slower stimuli, and then ran a staircase procedure to estimate their ISI thresholds. The staircase was run in blocks of 20 trials, alternating between single-task top and bottom conditions (details on staircase procedure available in White et al., 2020). During the main experimental blocks (20 trials each), blocks were run in a random order in sets of four, each set containing two dual-task blocks, one single-task top, and one single-task bottom. Testing sessions continued until each subject had completed approximately 60 blocks (1,200 trials, 600 of which were dual task).

The ISIs were initially set to the staircase threshold estimated and then adjusted to keep focused attention accuracy between 70% and 90% correct. Any run of four or more blocks within one session with an ISI that was either too high in single-task accuracy (>90%) or too low (<70%) was discarded and re-run. We ensured that the ISIs did not differ between dual- and single-task conditions in each session. The mean ISI in usable trials was 84 ms (*SEM* = 7 ms, range: 50–121 ms).

#### Analysis

Trials with fixation breaks (deviations in gaze position more than 1° from the central point during the stimulus presentation) were excluded from analysis (16% of trials on average). We measured accuracy as the area under the ROC Curve (A_g_), a signal-detection metric that takes into account the confidence level reported on each trial (Pollack & Hsieh, 1969). A_g_ can be considered a bias-corrected proportion correct, ranging from 0.5 (guessing) to 1.0 (perfect accuracy). For more details, see White et al. (2020).

Throughout the text, we report bootstrapped 95% confidence intervals (CIs) for average measurements. To compute these, we generated a distribution of 1,000 resampled means. Finally, we supplement our pairwise tests with Bayes factors (BFs), which quantify the strength of evidence. The BF is the ratio of the probability of the data under the alternate hypothesis (that two means differ) relative to the probability of the data under the null hypothesis (that there is no difference). A BF of 10 would indicate that the data are 10 times more likely under the alternate hypothesis than the null. BFs between 3 and 10 are regarded as substantial evidence for the alternate hypothesis, and BFs greater than 10 as strong evidence. Conversely, BFs between 1/3 and 1/10 are considered substantial evidence for the null hypothesis. We computed BFs for pairwise *t* tests and analyses of variance (ANOVAs) using the bayesFactor toolbox by Bart Krekelberg (10.5281/zenodo.4394422).

### Experiment 2

All stimuli, procedures, and analysis steps were identical to Experiment 1, except as described here.

#### Participants

Ten volunteers (seven female, one male, two nonbinary; 10 right-handed; age 20.6 ± 1.6 years) with normal or corrected-to-normal visual acuity participated. Three participants also completed Experiment 1.

#### Stimuli and procedure

The stimulus set was composed of 1,580 unique letter strings, half pronounceable and half unpronounceable (see Fig. 1B for examples). All letter strings in both categories contained four consonants and one vowel and had no meaning in English. The pronounceable strings were five-letter pronounceable pseudowords generated by the MCWord database to have bigram and trigram statistics matched to real words (Medler & Binder, 2005). The unpronounceable strings were selected from the “vowel present” stimulus set used in Experiment 1 and were unpronounceable based on the rules of English spelling. We then selected stimuli such that there was an equal number of strings with the same starting letter in each category.

The task was to report whether the postcued letter string was pronounceable or not. As in Experiment 1, participants pressed one of four keys for each postcued side. In order from left to right, the keys represented “sure unpronounceable,” “guess unpronounceable,” “guess pronounceable,” and “sure pronounceable”. Each subject completed approximately 60 blocks (1,200 trials). The mean threshold ISI between the stimuli and postmasks was 66 ms (*SEM* = 11 ms, range: 29–138 ms).

### Results

#### Attention operating characteristics

**Fig. 2:**
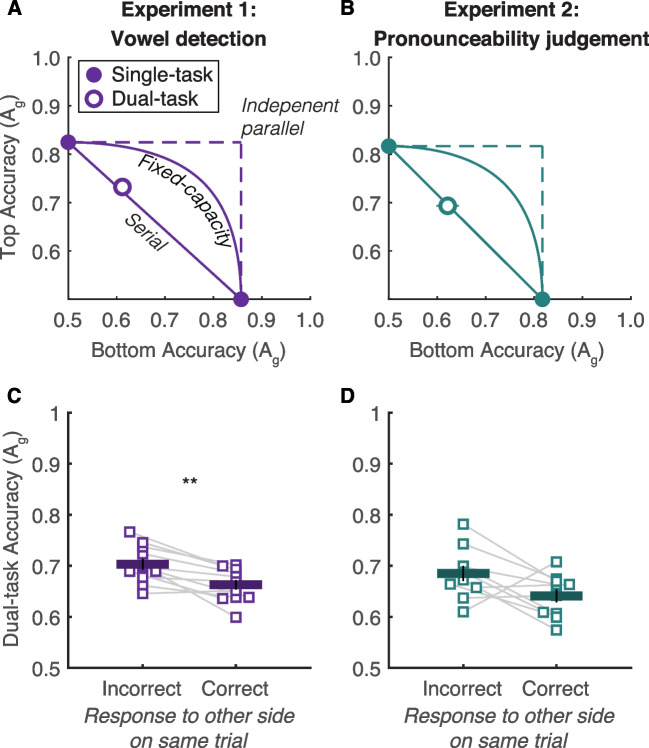
Attention operating characteristics (AOCs) and stimulus processing trade-offs. **A–B** AOCs constructed from mean accuracy (area under the ROC curve, A_g_) in Experiments 1 (left) and 2 (right). We compare the dual-task accuracy (open symbol) with the predictions of three models of processing capacity limits: independent parallel, fixed-capacity parallel, and all-or-none serial. In both experiments, dual-task accuracy is significantly worse than the fixed-capacity parallel model and indistinguishable from the serial model. Error bars indicating ±1 *SEM* are smaller than most of the data points. **C–D** Stimulus processing trade-offs in Experiments 1 (left) and 2 (right). We compare accuracy in two sets of dual-task responses—those in which the response to the *other* side on the same trial was incorrect, and those in which the response to the other side on the same trial was correct. Thin gray lines connect data points from the same individual participant. The horizontal positions of data points are jittered to avoid total overlap, but points from the same participant have the same relative jitter. The thick horizontal lines represent the means, with ±1 *SEM* error bars

The attention operating characteristic (AOC) is a plot that visualizes the difference in accuracy between single-task (focused attention) and dual-task (divided attention) conditions. Most importantly, we can visualize the quantitative predictions of specific models on the same plot (Sperling & Melchner, 1978). Figure 2A–B shows AOCs constructed from mean accuracy. The horizontal axis represents accuracy for the bottom stimulus and the vertical axis accuracy for the top stimulus. Single-task accuracies are pinned to their respective axes (filled circles). Dual-task accuracy forms a single point (open circle) in the 2-D space. Also shown are three model predictions: all-or-none serial processing, fixed-capacity parallel processing, and independent parallel processing. Details on each model are available in White et al. (2020). Note that the independent parallel model (Popovkina et al., 2021) has also been called the “unlimited capacity” model, to distinguish it from the fixed-capacity model (which assumes the two stimuli are not processed independently because they must share resources).

To compare accuracy to the model predictions, we constructed AOCs for each individual participant (shown in Supplementary Fig. 1). For each subject, we calculated the minimum distances between their dual-task accuracy point and the model predictions. We then estimated whether the average distances were distinguishable from zero.

##### Experiment 1

Mean dual-task accuracy fell on top of the all-or-none serial processing model’s prediction (Fig. 2A). The average distance from the line was 0.005 (±1 *SEM* = 0.031), which was not significantly above zero: *t*(10) = 0.47, *p* = 0.65, CI [−0.012 0.023], BF = 0.33. The average distance from the closest point on the fixed capacity parallel curve was 0.082 (±0.042), which was significantly different than zero: *t*(10) = 6.51, *p* < 10^-4^, CI [0.06 0.11], BF = 399. Thus, we reject the fixed-capacity parallel model and accept the serial model.

Interestingly, accuracies were not equal across the two sides (top vs. bottom), but the asymmetry differed across cue conditions—single versus dual task; interaction *F*(1, 40) = 68.7, *p* < 10^-9^). In the single-task condition, accuracy was slightly but not significantly better on the bottom side (mean difference = 0.03 ± 0.05 A_g_), *t*(10) = 2.05, *p* = 0.067, CI [0.008, 0.065], BF = 1.4). That is consistent with generally more acute vision in the lower visual field (Himmelberg et al., 2023). But in the dual-task condition, accuracy was better on the top side (mean difference = 0.12 ± 0.09), *t*(10) = 4.11, *p* = 0.002, CI [0.05, 0.162], BF = 21.6. Based on the position of each participant’s dual-task point along the serial model line, we could estimate this bias as a preference to process the top stimulus on some trials and the bottom on others. On average, participants processed the top stimulus on 74% of dual-task trials ± 3%, and the bottom stimulus on the remaining 26%. That is consistent with a bias to start “reading” from the top.

We also found that within dual-task trials, accuracy was on average slightly higher on the first response than the second response (mean difference = 0.028 ± 0.028 A_g_), *t*(10) = 3.11, *p* = 0.011; CI [0.049, 0.013]; BF = 5.6. This raises a concern that the dual-task deficit could be due to a limit on memory rather than on stimulus encoding. To address this concern, we reanalyzed the data including only the first response on all dual-task trials. The results were unchanged: We could still reject the fixed-capacity parallel model, but not the serial model.

##### Experiment 2

Mean dual-task accuracy fell on top of the all-or-none serial processing model’s prediction (Fig. 2B). The average distance from the line was −0.003 (±0.01), which was not significantly above zero: *t*(9) = 0.27, *p* = 0.79, CI [−0.022, 0.016], BF = 0.32. The average distance from the closest point on the fixed capacity parallel processing curve was 0.089 (±0.012), which was significantly different than zero: *t*(9) = 7.23, *p* < 10^-4^, CI [−0.112, −0.067], BF = 526. As in Experiment 1, therefore, we reject the parallel models and accept the serial model.

The asymmetries across sides were not as strong as in Experiment 1, but they did interact with cue condition, *F*(1, 36) = 5.08, *p* = 0.03. Accuracy was equivalent across sides in the single-task condition, *t*(9) < 1, *p* = 0.97, CI [−0.04, 0.04], BF = 0.31, and tended to be better on the top in the dual-task condition (mean difference = 0.07 ± 0.11), *t*(9) = 1.94, *p* = 0.08, CI [0.01, 0.13], BF = 1.2. According to the placement of each dual-task point along the serial model prediction line, participants processed the top stimulus on an average of 66% of trials (±6%), and the bottom stimulus on the remaining 34%. That is again consistent with a bias to process the top stimulus when both stimuli cannot be processed simultaneously (as when reading a list from top to bottom). But that bias can be voluntarily counteracted when the bottom stimulus is cued as the single focus of attention.

As in Experiment 1, accuracy on dual-task trials was slightly higher for the first response than second response (mean difference = 0.021 ± 0.034 A_g_), but not significantly so, *t*(9) = 1.83, *p* = 0.10; CI [0.00, 0.043]; BF = 1.05. When we re-analyzed the data including only the first response on all dual-task trials, we again came to the same conclusion: the serial model fits the data best.

#### Stimulus processing trade-offs

The all-or-none serial processing model assumes that only one stimulus can be processed per trial and no information about the other stimulus is acquired. With the assumption that the participant processes the top stimulus on some dual-task trials and the bottom stimulus on others, the model predicts a negative correlation between the accuracies of the two responses on each trial. Therefore, the participant is more likely to be correct about one side when they are incorrect about the other side. That is called a “stimulus processing trade-off” (White et al., 2018, 2020), and is predicted by the same serial model that predicts the diagonal line on the AOC (Fig. 2A–B). Note that this serial model assumes that the participant *never* processes two stimuli in the same trial.

To estimate the stimulus processing trade-off for each participant, we sorted all the dual-task responses into two sets, one in which the response to the *other side* on the same trial was correct, and one in which the response to the other side on the same trial was incorrect. Then we compared accuracy (A_g_) in those two sets of trials. In prior work, we have found this analysis to be more sensitive than simply computing the correlation coefficient between response accuracies, in part because A_g_ is a measure of accuracy that corrects for bias. See White et al. (2018).

##### Experiment 1

Figure 2C shows that accuracy in the dual-task condition was significantly higher when the response for the other stimulus was incorrect compared with when the response for the other stimulus was correct. The mean difference (in units of A_g_) was 0.040 (±0.01), *t*(10) = 4.51, *p* = 0.001, CI [0.024, 0.059], BF = 37. This result supports the serial model.

##### Experiment 2

Figure 2D shows that on average, the stimulus processing trade-off was as large as in Experiment 1, but it was not quite statistically significant because there was more variability across participants. The mean difference was 0.044 (±0.02), *t*(9) = 2.15, *p* = 0.06, 95% CI [−0.003 0.074], BF = 1.54.

To integrate data from both experiments, we fit a linear mixed-effects model that predicted accuracy as a function of experiment (1 vs. 2) and accuracy for the other stimulus on the same trial (correct vs. incorrect). There was a main effect of the other side’s accuracy, *F*(1, 38) = 16.8, *p* < 0.001, BF = 169, which did not differ across experiments, *F*(1, 38) = 0.03, *p* = 0.85, BF = 0.27. Thus, the stimulus processing trade-off is a robust phenomenon.

**Fig. 3:**
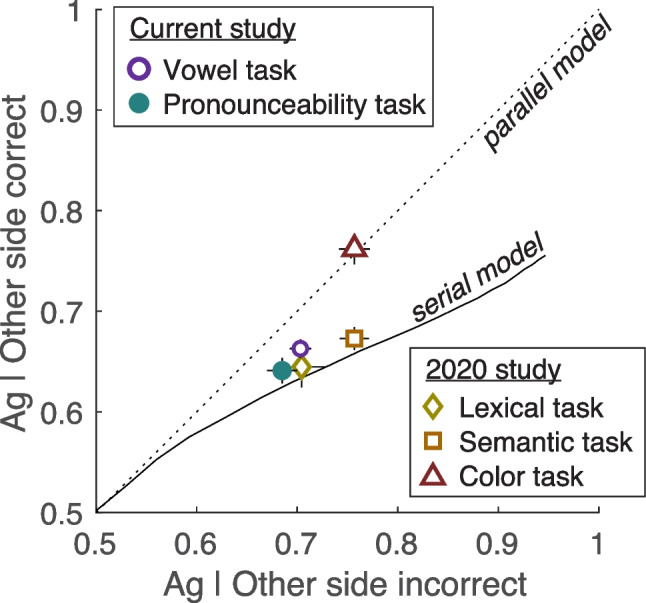
Stimulus processing trade-offs compared with model predictions and other tasks. Mean accuracy in the dual-task condition according to whether the response to the stimulus on the other side was incorrect (*x*-axis) versus correct (*y*-axis). The dotted diagonal line is the prediction of the fixed-capacity parallel model (no trade-off). The curved solid line is the prediction of the all-or-none serial model, generated by varying single-task discriminability. The data points labeled “2020 study” are from White et al. (2020)

We can also compare these effects to the predictions of the serial model and fixed-capacity parallel model, and to prior experiments. Figure 3 plots dual-task accuracy on trials when the other side was judged incorrectly on the *x*-axis, and accuracy when the other side was judged correctly on the *y*-axis. The prediction of the fixed-capacity parallel model is the dashed line with slope = 1 (no effect). The prediction of the all-or-none serial model is the solid black line, simulated for a range of single-task accuracy levels. See the Appendix of White et al. (2020) for details of this simulation.

Figure 3 shows that as the task gets easier overall, the stimulus processing trade-off effect predicted by the serial model gets larger. Specifically, the model’s prediction deviates further from the identity line as we move from left to right; as single-task discriminability increases, there is a bigger accuracy loss on dual-task trials when the other side is judged correctly. This is because we are assessing the difference between not processing a stimulus at all (because the other side got processed) and processing it as well as possible. That difference creates a bigger trade-off effect in the data as the discriminability of each individual stimulus increases, meaning that the probability of a correct response increases for stimuli that *do* get processed. (The probability of a correct response for stimuli that did not get processed is always 0.5.) In contrast, when each stimulus is harder to perceive (even with focused attention), the difference in the probability of a correct response between stimuli that do get processed and stimuli that do not get processed at all is smaller, thus the serial model predicts a smaller stimulus processing trade-off.

Mean data from the current study are the purple and green circles. These stimulus processing trade-offs are not quite as large as predicted by the serial model, but clearly deviate from the parallel model. Also shown are data from three different tasks collected in White et al. (2020): a lexical decision task, a semantic categorization task, and a color detection task. The data points for the present study (vowel and pronounceability task) are similar to those from the lexical task. Given the consistently serial result on the AOCs (Fig. 2A–B), we predict that had the tasks been slightly easier (e.g., with slightly bigger font, perhaps), the stimulus processing trade-offs would have been larger (as in the semantic task, represented by the orange square).

Given that the serial model predicts a small negative stimulus processing trade-off (at this level of overall accuracy), we consider the AOCs to be a more powerful analysis. In the AOCs, the serial model predicts a very large effect (of dividing compared with focusing attention) that can be distinguished from parallel models with greater statistical power. That is in fact what we found: the dual-task deficit was consistent with the serial model and ruled out both parallel models. The stimulus processing trade-offs provide converging evidence.

### Congruency effects

In the final analysis, we investigate how the response to one side depends on the stimulus that was on the other side. This was a planned analysis that we have applied to several previous experiments (White et al., 2018, 2020). “Congruent” trials are those in which the stimuli on both sides belong to the same category, so the same response would be correct for both sides. “Incongruent” trials are those in which the two stimuli belong to different categories, and the correct responses are different. In general, we expect higher accuracy on congruent trials (Eriksen & Eriksen, 1974; White et al., 2020). We analyzed these two sets of trials and fitted them with linear mixed-effect models, with cue condition (single-task vs. dual-task) as an interacting fixed effect. The results are in Supplementary Fig. S2.

#### Experiment 1

There was no main effect of congruency, *F*(1, 40) = 1.66, *p* = 0.20, BF = 0.36, nor interaction with cue condition, *F*(1, 40) = 1.28, *p* = 0.26, BF = 0.46.

#### Experiment 2

The congruency effect was larger in Experiment 2 than in Experiment 1, and statistically significant, *F*(1, 36) = 26.2, *p* = 10^-5^, BF = 2725. It did not vary across cue conditions, *F*(1, 36) = 0.04, *p* = 0.84, BF = 0.29. The mean congruency effect was 0.06 in Ag units (in both single- and dual-task trials).

Higher accuracy on congruent trials could arise for at least two reasons. The first is “cross-talk” that occurs while two stimuli are processed in parallel (Logan & Gordon, 2001). The second is selection errors: the participant either fails to attend to the precued stimulus, or confuses the two stimulus locations and reports what was presented at the side opposite the postcue (Palmer & Moore, 2009). Such selection errors have no effect on congruent trials but cause errors on incongruent trials.

The congruency effect in Experiment 2 is somewhat surprising. There should be zero congruency effect if the serial model is a valid approximation, *and* participants successfully attend to the precued side on every trial. The deficit of about 0.06 A_g_ on incongruent trials could arise if, on roughly 7% of single-task trials, participants attended to and reported the wrong side. The congruency effect in dual-task trials could arise if participants confuse the sides in about 10% of trials. That is not implausible.

## Discussion

We investigated the processing capacity limits that constrain letter identification and pronounceability judgments when attention is divided over two letter strings. We found large costs of dividing attention in both tasks, supporting the all-or-none serial processing model. For all individual participants, accuracy in the dual-task condition was so much worse than in the single-task condition that it best matched the serial model’s prediction (see Fig. S1). Also, accuracy for each side was impaired on trials when the other stimulus was judged correctly (although that was not quite statistically significant in Experiment 2). Both results support the existence of a bottleneck that allows letter identification and phonological decoding to occur for only one letter string at a time.

The large cost of dividing attention is likely due to the limited amount of time available to process each letter string. Postmasks controlled the amount of time available to process stimuli by clearing iconic memory after the ISI. A previous study *without* postmasks demonstrated results consistent with fixed-capacity parallel processing in a semantic search task (Scharff et al., 2011). One interpretation is that when visual information is presented for long durations or lingers in iconic memory, participants can process two stimuli sequentially within the span of one trial. For a related discussion in the context of object recognition, see a recent study by Popovkina et al. (2021).

Altogether, these results are remarkably similar to previous dual-task experiments with semantic categorization and lexical decision tasks (White et al., 2018, 2020; White, Palmer, et al., 2019b). See also Johnson et al. (2022) for consistent evidence using a partially valid cueing paradigm. Two prior dual-task studies also found performance consistent with the *parallel* model when the task was to judge the color of the letters (colored vs. gray) rather than word meaning (White et al., 2018, 2020). That is evidence that the capacity limits for written words vary depending on the depth of processing required by the task.

In the experiments presented here, which also required participants to process the letter strings at a sub-lexical level, we *could* have found performance consistent with parallel processing. We did not, and therefore conclude that the processing capacity limit constrains the ability to recognize individual letters within strings, and to judge the pronounceability or orthographic regularity of the strings. This suggests that focused attention is required for semantic categorization, letter identification, and phonological decoding of written words.

An important question is whether the two tasks utilized here tap into sub-lexical processes also involved in recognizing whole words, and thus inform at which stage of word recognition the “bottleneck” arises. Letter identification is a necessary step to word recognition (Pelli et al., 2003). The vowel detection task (Experiment 1) was designed to assess how well letters can be identified in two strings at once. While vowel detection per se is probably not a required process for higher-level word recognition tasks, this task serves as a proxy for the more general function of letter identification. Similarly, our pronounceability task was a proxy for phonological processing that is known to play a role in word recognition (Grainger et al., 2016). Nonetheless, according to the “dual-route” model of word recognition (Coltheart et al., 2001) skilled readers can extract semantic information directly from familiar visual word forms without an intermediate stage of phonological decoding. That may mean that phonological judgements are not relevant for skilled reading. That said, our phonological task essentially required the participant to judge the orthographic regularity or legality of letter strings (e.g., *crult* vs. *crtul*). Orthographic regularity is how well a letter string conforms to the statistical regularities of letter combinations, which impacts the print-to-sound mapping. Reading behavior and word recognition performance are sensitive to orthographic regularity (Chetail, 2017; Radach et al., 2004). Thus, we argue that these tasks are informative as to the capacity limits in word recognition, but additional work on this question is necessary.

Another possible limitation of our study arises from the fact that the letter strings were not words (which we chose to isolate sub-lexical processes). The word superiority effect shows that letters within words are reported more accurately than letters in nonwords (Riecher, 1969; Wheeler, 1970). This effect could be due to feedback connections between word representations and letter representations. An interesting question for future research is whether such interactive representations allow for parallel processing of letter identification if the stimuli were words rather than nonword letter strings.

We also acknowledge that the conditions in this study clearly differed from natural reading. English readers make a sequence of left-to-right saccades to place successive words in the high-resolution fovea (Rayner, 2009). In this study, participants fixated on a central point between two letter strings that were arranged vertically. It is possible that the reading circuitry is adapted to favor a horizontal flow of text. Thus, the capacity limit in these particular tasks may be less severe if the letter strings were placed to the left and right of fixation.

The position of our stimuli, above and below fixation, also affects what links we can draw between these behavioral data and an fMRI study that recorded activity in sub-regions of the “visual word form area” (VWFA) while participants made semantic judgments of words positioned left/right of fixation (White, Palmer, et al., 2019b). While activity in the anterior VWFA subregion was consistent with serial processing of one word at a time (as was task accuracy), activity in posterior VWFA was consistent with parallel processing of the two words. If we believe that the posterior VWFA processes letter strings at a sub-lexical level (Vinckier et al., 2007; Woolnough et al., 2021), then we would predict performance in the letter identification task—and perhaps even the phonological task—to be consistent with parallel processing as well. But that may only be the case when the stimuli are positioned left and right of fixation. That is a topic of future research. We note, however, that our present results are consistent with a more naturalistic experiment conducted by Reichle et al. (2008): Participants searched lists of words arranged horizontally (as in typical text), and were free to move their eyes. When the targets were specific letters, words with a specific rhyme, or words belonging to a semantic category, search times increased steeply as the number of words increased. Like the results we presented above, these data were consistent with serial processing of words for identifying letters and phonemes, as well as meaning.

In sum, we conclude that the bottleneck that constrains word recognition—at least under the specific conditions we have tested—occurs at a sub-lexical level. We found that attention must be focused in order to efficiently process letter strings even for sub-lexical tasks that require identification of parts of a word rather than the whole word itself. Future work is needed to determine at what stage, “below” the level of letter identification, the capacity limit arises, and to apply these questions to conditions more like natural reading.

### Supplementary information

ESM 1 (DOCX 743 kb)

## Data Availability

All the raw data collected for this study and the code to analyze are available Open Science Framework (10.17605/OSF.IO/RQZ8V).

## References

[CR1] Brainard, D. H. (1997). The psychophysics toolbox. *Spatial Vision*, *10*, 443–446. 9176952

[CR2] Chetail F (2017). What do we do with what we learn? Statistical learning of orthographic regularities impacts written word processing. Cognition.

[CR3] Coltheart M, Rastle K, Perry C, Langdon R, Ziegler J (2001). DRC: A dual route cascaded model of visual word recognition and reading aloud. Psychological Teview.

[CR4] Engbert R, Nuthmann A, Richter EM, Kliegl R (2005). SWIFT: A dynamical model of saccade generation during reading. Psychological Review.

[CR5] Eriksen BA, Eriksen CW (1974). Effects of noise letters upon the identification of a target letter in a nonsearch task. Perception & Psychophysics.

[CR6] Grainger J, Dufau S, Ziegler JC (2016). A vision of reading. Trends in Cognitive Sciences.

[CR7] Himmelberg MM, Winawer J, Carrasco M (2023). Polar angle asymmetries in visual perception and neural architecture. Trends in Neurosciences.

[CR8] Janini, D., Hamblin, C., Deza, A., & Konkle, T. (2021). General object-based features account for letter perception. *BioRxiv.*10.1101/2021.04.21.440772 10.1371/journal.pcbi.1010522 PMC9536565 36155642

[CR9] Johnson ML, Palmer J, Moore CM, Boynton GM (2022). Evidence from partially valid cueing that words are processed serially. Psychonomic Bulletin & Review.

[CR10] Legge, G. E., Ahn, S. J., Klitz, T. S., & Luebker, A. (1997). Psychophysics of reading—XVI. The visual span in normal and low vision. *Vision Research*, *37*(14), 1999–2010. 10.1016/S0042-6989(97)00017-5 10.1016/s0042-6989(97)00017-5 9274784

[CR11] Logan GD, Gordon RD (2001). Executive control of visual attention in dual-task situations. Psychological Review.

[CR12] Medler, D. A., & Binder, J. R. (2005). *MCWord: An on-line orthographic database of the English language.* http://www.neuro.mcw.edu/mcword/. Accessed 2021.

[CR13] Palmer J, Moore CM (2009). Using a filtering task to measure the spatial extent of selective attention. Vision Research.

[CR14] Pelli DG (1997). The VideoToolbox software for visual psychophysics: Transforming numbers into movies. Spatial Vision.

[CR15] Pelli DG, Farell B, Moore DC (2003). The remarkable inefficiency of word recognition. Nature.

[CR16] Pelli DG, Tillman KA, Freeman J, Su M, Berger TD, Majaj NJ (2007). Crowding and eccentricity determine reading rate. Journal of Vision.

[CR17] Pollack I, Hsieh R (1969). Sampling variability of the area under the ROC-curve and of d′e. Psychological Bulletin.

[CR18] Popovkina, D. V., Palmer, J., Moore, C. M., & Boynton, G. M. (2021). Is there a serial bottleneck in visual object recognition? *Journal of Vision*, *21*(3):15, 1–21. 10.1167/jov.21.3.15 PMC7961120 33704373

[CR19] Radach R, Inhoff A, Heller D (2004). Orthographic regularity gradually modulates saccade amplitudes in reading. European Journal of Cognitive Psychology.

[CR20] Rayner K (2009). Eye movements and attention in reading, scene perception, and visual search. Quarterly Journal of Experimental Psychology.

[CR21] Rayner, K., Schotter, E. R., Masson, M. E. J., Potter, M. C., & Treiman, R. (2016). So much to read, so little time: How do we read, and can speed reading help? *Psychological Science in the Public Interest, 17*(1). 10.1177/1529100615623267 10.1177/1529100615623267 26769745

[CR22] Reichle ED, Liversedge SP, Pollatsek A, Rayner K (2009). Encoding multiple words simultaneously in reading is implausible. Trends in Cognitive Sciences.

[CR23] Reichle ED, Pollatsek A, Rayner K (2006). E-Z Reader: A cognitive-control, serial-attention model of eye-movement behavior during reading. Cognitive Systems Research.

[CR24] Reichle ED, Vanyukov PM, Laurent PA, Warren T (2008). Serial or parallel? Using depth-of-processing to examine attention allocation during reading. Vision Research.

[CR25] Riecher GM (1969). Perceptual recognition as a function of meaningfulness of stimulus material. Journal of Experimental Psychology.

[CR26] Scharff A, Palmer J, Moore CM (2011). Extending the simultaneous-sequential paradigm to measure perceptual capacity for features and words. Journal of Experimental Psychology: Human Perception and Performance.

[CR27] Shaw, M. L. (1980). Identifying attentional and decision-making components in information processing. *Attention and performance VIII* (pp. 277–295). Psychology Press.

[CR28] Snell J, van Leipsig S, Grainger J, Meeter M (2018). OB1-reader: A model of word recognition and eye movements in text reading. Psychological Review.

[CR29] Sperling G, Melchner MJ (1978). The attention operating characteristic: Examples from visual search. Science.

[CR30] Veldre A, Reichle ED, Yu L, Andrews S (2023). Lexical processing across the visual field. Journal of Experimental Psychology: Human Perception and Performance.

[CR31] Vidal C, Content A, Chetail F (2017). BACS: The Brussels Artificial Character Sets for studies in cognitive psychology and neuroscience. Behavior Research Methods.

[CR32] Vinckier F, Dehaene S, Jobert A, Dubus JP, Sigman M (2007). Hierarchical coding of letter strings in the ventral stream: Dissecting the inner organization of the visual word-form system. Neuron.

[CR33] Wheeler DD (1970). Processes in word recognition. Cognitive Psychology.

[CR34] White AL, Boynton GM, Yeatman JD (2019). You can’t recognize two words simultaneously. Trends in Cognitive Sciences.

[CR35] White AL, Palmer J, Boynton GM (2018). Evidence of serial processing in visual word recognition. Psychological Science.

[CR36] White AL, Palmer J, Boynton GM (2020). Visual word recognition: Evidence for a serial bottleneck in lexical access. Attention, Perception, and Psychophysics.

[CR37] White AL, Palmer J, Boynton GM, Yeatman JD (2019). Parallel spatial channels converge at a bottleneck in anterior word-selective cortex. Proceedings of the National Academy of Sciences.

[CR38] White, A. L., Runeson, E., Palmer, J., Ernst, Z. R., & Boynton, G. M. (2017). Evidence for unlimited capacity processing of simple features in visual cortex. *Journal of Vision*, *17*(6):19, 1–20. 10.1167/17.6.19.doi 10.1167/17.6.19 PMC5488877 28654964

[CR39] Woolnough O, Donos C, Rollo PS, Forseth KJ, Lakretz Y, Crone NE, Fischer-baum S, Dehaene S, Tandon N (2021). Spatiotemporal dynamics of orthographic and lexical processing in the ventral visual pathway. Nature Human Behaviour.

[CR40] Yeatman JD, White AL (2021). Reading: The confluence of vision and language. Annual Review of Vision Science.

