# Supplementary material for: Severe processing capacity limits for sub-lexical features of letter strings
Source: Atten Percept Psychophys. 2024 Jan 3;86(2):643–52. doi: 10.3758/s13414-023-02830-1 (PMC10805793; doi:10.3758/s13414-023-02830-1)
Supplement: Supplementary file 1 — (DOCX 743 kb) [file 13414_2023_2830_MOESM1_ESM.docx]

**Supplementary materials for**

***Severe processing capacity limits for sub-lexical features of letter strings***

by Campbell, Oppenheimer, White

**Figure S1:** Individual participant attention operating characteristics in both experiments. Solid points are single-task accuracy levels (in units of A_g_), and open points are dual-task accuracy levels. Model predictions as in Figure 2A.

**Figure S2:** Congruency effects on accuracy in each experiment, separately for single-task and dual-task trials. “Congruent” trials are when the stimuli on both sides belong to the same category. “Incongruent” trials are when the two stimuli belong to different categories. Asterisks indicate significant congruency effects within each cue condition (*p<0.05, ***p<0.001). Format as in Figure 2C.
